# Automated Laboratory Security Tiers: a framework for evaluating and mitigating biosecurity risks from latent capabilities

**DOI:** 10.3389/fmicb.2026.1832401

**Published:** 2026-07-15

**Authors:** Matthew D. Smith, Moritz S. Hanke, Rebecca L. Moritz, David R. Gillum, Jaspreet Pannu

**Affiliations:** 1 Center for Health Security, Johns Hopkins Bloomberg School of Public Health, Johns Hopkins University, Baltimore, MD, United States; 2 Tutela Strategies, LLC, Reno, NV, United States; 3 Compliance and Research Administration, University of Nevada, Reno, Reno, NV, United States

**Keywords:** automated biological laboratories, biosecurity, cloud laboratories, cyberbiosecurity, laboratory automation, viral rescue

## Abstract

Automated biological laboratories are a growing accelerator of scientific research, enabling remote execution through advanced robotics and computational systems. Automation is deployed across a range of settings, including modular automation platforms, centralized biofoundries, and cloud laboratories. For the most highly automated of these, the defining characteristics include remote operation, limited human oversight, and integrated automation. These technologies might enable fully autonomous biological research, also referred to as “closed-loop” discovery or self-driving laboratories. They can also introduce new vulnerabilities that existing biosafety and biosecurity frameworks do not sufficiently address. We first map the current landscape of automated biological laboratories, define what distinguishes them from conventional laboratories, and argue that existing biosafety and biosecurity oversight fails to account for risks from the latent capabilities of these laboratories. We then analyze multiple threat vectors, including malicious orders, insider threats, and cyberattacks. Through these vectors, an actor may be able to compromise laboratory infrastructure and orchestrate pathogen synthesis or protein engineering, without the knowledge of laboratory operators. We propose the Automated Laboratory Security Tier (AST) framework to categorize facilities into three tiers based on the biosecurity risks they could pose if fully compromised. We use “fully compromised” to mean an attacker can control protocol execution and relevant information technology and operational technology (IT/OT) controls sufficiently to bypass all oversight and safeguards. We outline methods to improve customer verification, order screening, cybersecurity measures, insider threat protections, and independent security assessments. Although limited information regarding automated laboratory capabilities is publicly available, we argue that most current automated laboratories do not possess latent capacity for serious harms. These risks appear low for most current facilities, but laboratories outside the scope of our review may possess greater latent capability, and capabilities may advance faster than anticipated. Our analysis draws on expert interviews with automated laboratory operators and biosecurity experts. We recommend that researchers and industry stakeholders engage with standard-setting bodies to establish security standards for automated biological laboratories. With ongoing monitoring of capability levels and adoption of proportionate safeguards, these laboratories can be safely operated.

## 1 Introduction

Automation could fundamentally reshape how biological research is conducted. Researchers can already design experimental protocols using software, submit them to remote facilities at scale, and receive digital results without ever handling biological materials directly (Arias and Taylor, 2024; Lee et al., 2025; Tobias and Wahab, 2025). These automated research laboratories, whether guided by human or AI commands, could significantly accelerate and scale research once their capabilities expand. Other benefits include increased reproducibility through standardized protocols, massive parallelization of experiments, reduced exposure risks for laboratory workers, and broader and more capital-efficient access to advanced instruments (Holland and Davies, 2020; Armer et al., 2023; Adam, 2024). The trajectory points to automated laboratories that are increasingly capable: first of accelerating human-led research, and potentially of driving research autonomously. As automated laboratory capabilities expand, they will likely accelerate discovery across fields from drug development, to materials science, to synthetic biology.

But capability cuts both ways. For example, a sufficiently skilled virologist can already synthesize horsepox, and by extension likely smallpox, from synthesized DNA fragments (Kupferschmidt, 2017; Noyce et al., 2018). A highly capable automated laboratory could potentially produce pathogens from unregulated starting materials: DNA nucleotides, cell lines, and standard reagents. In addition, an automated laboratory could optimize the properties of pathogen-relevant proteins, such as receptor binding affinity or immune evasion, through directed evolution workflows (Zhang et al., 2025; Wang et al., 2026). Addressing these and other potential biosecurity risks is what clears the path to the enormous benefits of automated laboratories.

We cover current U.S. regulatory frameworks in section “2.4 Existing regulatory frameworks,” with a full inventory of relevant U.S. regulations in Supplementary Material 2. Existing biosafety frameworks and regulations do not account for what automated laboratories could produce if compromised (CDC, 2020; NIH, 2024). To address this gap, we review what automated laboratories can do today, lay out the most pressing threat vectors, and propose the Automated Laboratory Security Tier (AST) framework, which scales security measures to match the latent capabilities of each laboratory. By latent capability we mean what a laboratory could produce if compromised, rather than what it currently handles.

Automated laboratories offer a potential biosecurity gain worth noting. Concentrating capability into a small number of monitored facilities makes consistent, uniform oversight more achievable than across a distributed landscape of smaller, manually operated laboratories. The AST framework proposes proportionate controls to defend automated laboratories against misuse.

The closest prior work on this threat model comes from RAND. A 2025 paper maps the global cloud laboratory landscape and analyzes how automated laboratories, including those focused on legitimate research, could be exploited by actors seeking chemical or biological weapons (Lee et al., 2025). This paper documents 15 cloud laboratory organizations across five countries, including publicly available data on facility size, scientific discipline, focus area, and instrument counts where available, and is the most detailed publicly available landscape mapping of cloud laboratory capabilities to date. A companion 2024 commentary proposes a Cloud Laboratory Security Consortium modeled on the IGSC, with shared screening, know-your-customer standards, and cross-laboratory data sharing (Lee and Del Castello, 2024). Building on these analyses, we formalize latent capability as a classification basis and propose a tiered framework that scales security requirements to that capability. Other relevant work covers the cyberattack surface of wet laboratory automation (Dalal et al., 2023) and AI-bio-automation integration risks more broadly (Wang et al., 2026). We also extend the threat model to insider threats and cyberattacks on laboratory control systems, with specific controls drawn from IEC 62443-3-3 (International Electrotechnical Commission, 2013) and NIST SP 800-160 (Ross et al., 2021; Stouffer et al., 2023).

Our analysis focuses on scenarios where human actors could compromise automated laboratories without operators’ knowledge, whether through malicious orders, insider access, or cyberattacks. The security controls we propose target the access vectors themselves and would apply regardless of whether compromise is orchestrated by a human or an AI system.

## 2 Mapping the automated laboratory landscape

### 2.1 Methodology

We performed a mixed-methods analysis based on expert interviews and qualitative desk research. Nineteen semi-structured interviews (45–60 min) were conducted from September to December 2025 with 21 participants, including biosecurity and related domain experts (nine participants), biotechnology industry professionals (seven participants), and automated laboratory operators and scientists (five participants, covering cloud laboratories, biofoundries, and other automated laboratory settings). We sought representation across these three groups to capture, respectively, the policy and threat-landscape perspective, industry practices in adjacent domains that might transfer to automated laboratories, and operational ground truth from automated laboratory personnel. Initial interviewees were identified through targeted outreach to known experts in biosecurity policy, automated laboratory operations, and adjacent fields. Subsequent participants were identified through snowball sampling, with introductions from earlier interviewees expanding coverage to organizations not on our initial outreach list.

The desk review covered publicly available materials on cloud laboratories and biofoundries, biosafety and biosecurity guidance, U.S. laws and regulations, as well as operational technology and cybersecurity standards. We anonymized interview sources and omitted or generalized sensitive operational details. Additional details on our interview approach are provided in Supplementary Material 1.

### 2.2 Defining automated biological laboratories

Existing definitions of automated biological laboratories do not adequately capture the operational diversity of systems such as biofoundries and cloud laboratories. For this analysis, we define an automated biological laboratory as a facility meeting three criteria:

1. Computational systems direct and coordinate biological experimental workflows.
2. Remote equipment operation is enabled through digital interfaces, allowing design, initiation, and monitoring of experiments without physical human presence.
3. Multiple automated instruments are integrated under computational control.

Together these criteria define the security profile that motivates this paper: computational direction creates the cyberattack surface, remote operation expands access beyond physical proximity, and multi-instrument integration potentially enables workflows such as pathogen synthesis that no single instrument could accomplish.

The definition is focused on laboratory systems, downstream of experimental design, and is agnostic to whether experiments are designed by a human or AI system. To qualify as an automated biological laboratory, human operators must primarily support machine-generated protocols and maintenance tasks rather than making independent experimental decisions. However, the humans and organizations operating these facilities remain accountable for biosafety, biosecurity, and other areas of risk and operations.

Automated biological research spans a spectrum of laboratory settings that differ in their degree of integration, scale, and capacity for remote operation. At the most basic level, many conventional laboratories now incorporate modular laboratory automation platforms which automate discrete tasks like pipetting, sample preparation, or assay execution. These systems are typically deployed as stand-alone units within otherwise human-operated laboratories, with researchers remaining physically present and responsible for experimental design, oversight, safety, security, waste disposal, and other operational tasks. These systems would not qualify under our definition of automated biological laboratories.

At a higher level of sophistication, centralized biofoundries integrate automation across much larger portions of the experimental workflow. These biofoundries operate at industrial scale, combining robotics, laboratory information management systems, and process engineering to support an iterative design-build-test-learn cycle (Chao et al., 2015; Holowko et al., 2021). This work may serve commercial partners or in-house research programs. In these settings, automation is tightly coupled across multiple stages of the pipeline, resembling a factory floor more than a traditional research laboratory, though human staff may still play a central role in supervision, decision-making, operations, and maintenance. Well-known examples include the Department of Energy Agile BioFoundry, the London Biofoundry at Imperial College London, and other members of the Global Biofoundries Alliance (Hillson et al., 2019). Some of these biofoundries qualify as automated biological laboratories, depending on their extent of computational direction, remote operation, and integration of automated systems.

At the most advanced end of the spectrum are commercial cloud laboratories, providing fully remote experimental execution as a service. In cloud laboratories, external users design experiments digitally and submit them through software interfaces, while on-site robotic systems and human workers carry out the physical work with minimal or no direct interaction with customers (Lee et al., 2025). According to our interviews and one cloud laboratory’s website (Emerald Cloud Lab, 2022, 2026), these laboratories routinely accept customer samples and, upon request, return experimental products to users. This enables researchers to conduct wet-laboratory experiments without ever setting foot in the laboratory itself. Emerald Cloud Lab exemplifies this fully remote model and meets all three criteria of our definition, as do other cloud laboratories.

Any governance or controls on automated laboratories must take into account the broad range of capabilities and models across these laboratories. Because of this, we propose a capability-based security framework.

### 2.3 Risks from latent capabilities

Historically, physical barriers have posed major obstacles to biological misuse. These include challenges in acquiring materials, developing tacit laboratory knowledge, and executing complex protocols (Sonia Ben Ouagrham-Gormley, 2014). Automated laboratories are intended to lower these barriers for beneficial purposes, but could also enable malicious actors unwilling or unable to conduct physical work themselves (Lee et al., 2025).

This shift creates a gap that existing frameworks do not address. A facility’s latent capability–what it could produce if compromised–can diverge sharply from what it knowingly handles. A facility working with low-risk organisms but containing nucleic acid synthesizers, synthetic biology capabilities, and cell culture systems may in the future possess a latent ability to synthesize functional viruses through viral rescue, or perform automated protein engineering to identify novel pathogen variants. Latent capability is the central analytical lens of this paper. Section “4 The Automated Laboratory Security Tier (AST) framework” develops the AST framework, which classifies facilities by latent capability rather than by what they currently handle.

Automation and remote operation expose these latent capabilities to the cyber domain. Unlike conventional laboratories, where harmful biological work would require physical presence and direct manipulation of equipment, automated biological laboratories present specific cyberattack surfaces: remote experimental submission allows anyone with network access to direct laboratory work; software-defined protocol execution means the same control systems running benign experiments can in principle be redirected toward harmful ones; and integrated workflows, where multiple instruments are coordinated under computational control, allow a single compromised system to trigger sequences that no single instrument could accomplish alone. Operational technology environments often have vulnerabilities (Dalal et al., 2023; Dragos Inc, 2026), attackers and defenders are in a constant arms race (Huntley and Shives, 2024; Office of the Director of National Intelligence, 2025), and even isolated computer networks can sometimes be compromised, albeit through extreme effort (Langner, 2011). Addressing these risks requires bringing together biosafety/biosecurity experts and cybersecurity professionals to secure automated laboratories, similar to other cyberbiosecurity efforts. Existing U.S. regulatory frameworks were not designed for either the latent capability problem or these cyber vulnerabilities, as the next section maps.

### 2.4 Existing regulatory frameworks

Our regulatory analysis focuses on the United States, though other jurisdictions have analogous pathogen classification and oversight systems. The U.S. has no single overarching biosafety and biosecurity law, “…beyond those in the Federal Select Agent Program, which covers only certain types of biological agents and toxins” (Kuiken, 2024). Instead, oversight relies on a patchwork of federal statutes, agency regulations, funding conditions, and voluntary guidance (Gillum, 2025). Cyberbiosecurity regulation is even less developed. No U.S. framework specifically addresses the cyber-physical risks of automated biological laboratories, and broader cybersecurity gaps in the bioeconomy have been flagged in recent assessments (National Academies of Sciences, Engineering, and Medicine, 2020; Guise et al., 2024). Automated laboratories are subject to legally binding federal regulations depending on the work they perform, including regulations from the Occupational Safety and Health Administration (OSHA), Food and Drug Administration (FDA), and Environmental Protection Agency (EPA), as well as Clinical Laboratory Improvement Amendments (CLIA) and export control regulations. See Supplementary Material 2 for a full regulatory inventory. There are three areas of U.S. oversight and guidance most relevant to the biosecurity risks we address here:

- The National Institutes of Health (NIH) Guidelines for Research Involving Recombinant or Synthetic Nucleic Acid Molecules (NIH, 2024), hereafter referred to as “NIH Guidelines.”
- The Centers for Disease Control and Prevention’s (CDC) Biosafety in Microbiological and Biomedical Laboratories (CDC, 2020), hereafter referred to as “BMBL.”
- The Federal Select Agent Program regulations (7 CFR Part 331, 9 CFR Part 121, and 42 CFR Part 73), covering human, animal, and plant pathogens and toxins, hereafter referred to as “Select Agents.”

These three focus on “…[protecting] laboratory workers, the environment, and the public from exposure to infectious microorganisms that are handled and stored in the laboratory” (CDC, 2020).

This is in contrast to the risks these laboratories could pose if compromised, a distinction that matters most for automated laboratories given their degree of automation and remote operation. Facilities with advanced synthetic biology capabilities but no regulated materials (e.g., Select Agent genetic elements, recombinant and/or synthetic nucleic acids, and recombinant and/or synthetic organisms) on-site fall outside existing biosecurity frameworks, even though customers may be able to ship materials to those facilities with limited oversight or verification. There are some permitting requirements when importing certain biological materials or transporting them across state lines in the United States, but a malicious actor would likely not follow these requirements.

### 2.5 Current state and trajectory

In interviews conducted for this study, respondents characterized current commercial automated laboratories as primarily supporting standardized, repeatable pharma and biotech workflows, rather than bespoke, high-complexity biological protocols. The actual capabilities for synthesizing harmful biological agents in commercial automated laboratories as of the end of 2025 are likely very limited, but have not been formally evaluated for these risks. This assessment comes with significant uncertainty. As capabilities advance, methods for standardized capability and risk evaluation are needed, with a particular focus on what these laboratories could produce if compromised.

Automated laboratory capabilities have arguably underperformed early projections. Predictions of a “robotic revolution” in life science research have historically outpaced reality (Holland and Davies, 2020), and critics continue to worry about hype (Adam, 2024). But this dynamic may change, and we believe security measures will be far easier to build in now than to retrofit after latent capabilities advance to concerning levels.

## 3 The threat environment

For automated laboratories to pose serious biosecurity threats from misuse, several factors must converge:

1. Capability: The laboratory must have sufficient technical capability to execute such protocols.
2. Access: Threat actors must gain access to laboratory systems through a malicious biological order, an insider, or a cyberattack, and security measures must fail to detect and prevent the attack (National Science Advisory Board for Biosecurity, 2009; Department of Homeland Security, 2013; Cummings et al., 2021).
3. Protocols: The threat actor must possess or obtain protocols for synthesizing harmful biological agents or optimizing pathogen-relevant proteins. Based on our interviews, protocols must be adapted to the target facility, but future advances may make this unnecessary.

The AST framework tiers laboratories by capability. Our proposed security controls, detailed in Table 1, address access through measures targeting each threat vector.

**TABLE 1:** Automated Laboratory Security Tier framework: capability definitions and required safeguards.

Tier		AST-0	AST-1	AST-2 Low	AST-2 High
**Laboratory classification criteria**		Not equipped to synthesize, modify, or propagate living organisms, viruses, or nucleic acids.	Equipped to synthesize, modify, or propagate living organisms, viruses, and/or nucleic acids. And/or: Capable of synthesizing organisms not associated with disease in healthy adult humans (RG1) if fully compromised.	Capable of synthesizing one or more human, animal, or plant pathogens classified as RG2-4 or designated as HHS or USDA Select Agent (excluding non-infectious toxins), if fully compromised. And/or: Capable of automated protein engineering, including directed evolution and high-throughput screening, on pathogen-relevant proteins, if fully compromised. Potential analogue tests: 1. Can the laboratory synthesize PR8, a mouse-adapted flu virus, as an analogue for RG2-3 influenza. 2. Can the laboratory synthesize horsepox, as an analogue for smallpox. 3. Can the laboratory optimize binding affinity of a benign protein/protein pair, as an analogue for pathogen-relevant protein engineering.	
**Safeguards for tier (cumulatively include safeguards of previous tiers)**	Customer identity checks	No additional safeguards required beyond standard practices.	Basic identity verification (e.g., institutional email verification).	Risk-based verification with watchlist and government checks, rigorous KYC (know your customer) process.	Shared alerts (between automated laboratory organizations) on suspicious orders/ customers; beneficial-ownership screening; payment tracing.
	Order and experiment screening	No additional safeguards required beyond standard practices.	Nucleic acid sequence screening (for incoming customer orders).	Protocol screening for dual-use techniques (e.g., viral rescue), advanced nucleic acid synthesis screening (e.g., functional homologue detection), sequencing of all incoming materials.	Sequencing of all outgoing materials before shipment (important for transport security requirements and bioagent shipment security).
	Cybersecurity and equipment protection	No additional safeguards required beyond standard practices.	Maintain IT/OT (information technology/operational technology) inventory; remove default credentials; require admin multi-factor authentication (MFA); patch systems; no public endpoints.	Strong authentication and network access controls (Zero Trust).	Rigorous security tailored specifically for laboratory equipment and automation aligned with IEC 62443-3-3 SL3–SL4 (International Electrotechnical Commission, 2013), including zone-based isolation of laboratory automation systems, hardened vendor components, and adversary-resilient design assumptions; isolation of laboratory information systems from the internet; regular resilience drills, e.g., National Institute of Standards and Technology (NIST) SP 800-160 Vol. 2 (Ross et al., 2021).
	Insider threat prevention	No additional safeguards required beyond standard practices.	No additional safeguards required beyond standard practices.	Thorough employee background checks updated regularly; cooperation with law enforcement.	Two-person oversight for critical tasks; continuous reliability monitoring, analogous to FSAP Tier 1 (Federal Select Agent Program, 2025).
	Independent security checks	No additional safeguards required beyond standard practices.	No additional safeguards required beyond standard practices.	Regular third-party audits; active red-team simulations.	Full-chain adversarial exercises: regulator- observed scenarios combining cyber, insider, and order vectors.

AST-2 Low vs. High designations are determined on a case-by-case basis by operational risk factors (see main text).

### 3.1 Primary threat vectors

Malicious biological orders: Threat actors could establish seemingly legitimate customer relationships and submit protocols that use the laboratory’s capabilities for pathogen-related tasks like viral rescue. Concerns about misuse of biological orders are not new; gene synthesis providers have faced this risk for years and developed norms for voluntary screening (Carter and Friedman, 2015; Diggans and Leproust, 2019). What is new for automated laboratories is the breadth of integrated capability behind a single customer relationship. An attacker could exploit a highly capable facility’s integrated capabilities to generate functional biological agents (Noyce et al., 2018; Xie et al., 2020). Sophisticated actors might obfuscate intent through decoy protocols, fragmented orders across multiple services, or obfuscated nucleic acid sequences. Experts with direct experience in gene synthesis and cloud laboratory biosecurity noted that most intermediate steps in viral rescue workflows are largely indistinguishable from standard laboratory operations, unlike gene synthesis screening where many sequences of concern are distinctive and identifiable. This makes protocol-level screening insufficient as the sole detection mechanism.

Customer-provided biological samples introduce another vulnerability. There are no existing requirements in the U.S. to sequence incoming materials to automated laboratories, creating opportunities to submit pathogenic genetic constructs disguised as benign materials. Among the U.S. federal regulations surveyed in Supplementary Material 2, none require sequencing of customer-supplied biological materials at automated laboratories. In our interviews with automated laboratory operators, we heard that they don’t currently sequence incoming materials, although this sequencing may be performed voluntarily by some organizations. Without thorough sequencing of incoming materials, laboratories could unknowingly amplify or synthesize harmful agents.

Gene synthesis providers and some contract research organizations face a related challenge in the form of customer-supplied physical material such as vectors for cloning, which is also typically not sequenced. The voluntary screening regime developed in the gene synthesis sector covers sequences ordered for synthesis, not physical samples sent to providers. Two factors make the automated laboratory case distinct: integrated capabilities can extend beyond synthesis and cloning to multi-step workflows including viral rescue, and the automated laboratory sector has not yet developed analogous voluntary screening norms.

Insider threats: These attacks leverage trusted employees’ legitimate access to create harmful biological agents (National Science Advisory Board for Biosecurity, 2009; Department of Homeland Security, 2013). An insider, especially with admin access to information technology (IT) and operational technology (OT) systems, could modify protocols, misdirect samples, or disable safety systems while appearing to perform routine duties.

Cyberattacks for biological misuse: Unlike conventional cyberattacks for data theft or ransom, these would seek to hijack laboratory control systems to produce biological agents or engineer pathogen-relevant proteins. Attackers could inject malicious protocols into legitimate workflows, alter synthesis parameters to create different products than intended, or compromise multiple systems to aggregate harmful capabilities. Even high-containment biological laboratories have vulnerabilities, and these vulnerabilities are sometimes published openly (Poste and Gillum, 2023). While the required capabilities for a full compromise are currently limited to highly capable cyber actors, and we heard in interviews that some cloud laboratories have strong cybersecurity, this threat vector may grow as laboratories increase connectivity and as offensive cyber tools become more powerful and accessible. In other interviews, experts noted that automated laboratory equipment has known cyber vulnerabilities, and that even well-resourced facilities may not have subjected their isolation controls to external adversarial testing.

### 3.2 Laboratory errors

Unlike the previous threat vectors, the category of laboratory errors does not involve actors trying to compromise the laboratory. While automation will likely reduce some types of human error, new categories of errors may emerge. Most will be harmless and recoverable, but some could cause serious harm. Biological toxins have legitimate research and commercial applications, such as botulinum toxin in pharmaceutical R&D and manufacturing, meaning automated laboratories may have valid reasons to work with them. However, automated systems could inadvertently produce harmful quantities of biological toxins through corrupted or erroneous protocols, such as unit conversion errors in protocol specifications. The quantity produced would depend on available reagents, equipment capacity, and the specific workflow, but it is feasible that an automated laboratory could produce dangerous quantities of many toxins. Existing safety measures such as quality control checks and personal protective equipment may mitigate some of these risks, but safety systems designed for intended production levels may not account for errors that dramatically exceed those levels.

### 3.3 Mitigating factors

Laboratory operators and biosecurity experts we interviewed raised several considerations that temper these risk assessments. We organize these into three groups: Technical limits, organizational and structural safeguards, and malicious actor dynamics.

#### 3.3.1 Technical limits

- Technical barriers to automation: Much biological laboratory work depends on tacit knowledge: recognizing subtle contamination, adjusting conditions based on cell morphology, or troubleshooting through intuition. Establishing complex protocols like viral rescue in automated systems currently requires considerable human intervention. However, AI systems designed to make more protocols automatable may reduce these barriers over time.
- Limited current synthetic biology capabilities: Many cloud laboratories focus on routine analytical work rather than synthetic biology. Whether and when these facilities will expand into higher-risk capabilities remains uncertain. However, unless applicable federal, state, or local laws restrict them, there are no clear technical or regulatory barriers that prevent automated laboratories from developing these capabilities.
- Protocol limitations: Feasible viral rescue protocols exist to recover many known viruses, and releases of wild-type or lightly mutated viruses could cause significant harm using established methods (Cello et al., 2002; Tumpey et al., 2005; Perez, 2017). Techniques that increase pathogenicity or transmissibility have been demonstrated in conventional research laboratories for years (Herfst et al., 2012; Imai et al., 2012). More speculative are scenarios involving *de novo* viral design or novel AI-generated protocols for enhanced function. However, recent studies found that AI systems can develop new molecular protocols, including novel enhanced methods for cloning and cell-free protein synthesis (Eroshenko et al., 2025; Smith et al., 2026). Emerging viral design methods, such as those shown for phages in one recent report (King et al., 2025) are beyond the scope of our analysis but warrant monitoring.

#### 3.3.2 Organizational and structural safeguards

- Human oversight as safeguard: Current facilities often employ staff that monitor protocol and equipment, and they would likely notice a new type of experiment such as viral rescue. This oversight may not scale as facilities grow more automated and handle higher volumes. Training of human workers could help, and is necessary for the safe operation of a complex automated lab, but the vision of automated laboratories is to minimize human work so research can scale.
- Existing incentives and safeguards: All corporations take steps to minimize their overall risk profile and avoid being the target of cyberattacks. Many go beyond what’s legally required to safeguard their reputations, profits, workers, and the communities in which they operate. While this is broadly true, even well-intentioned, well-resourced, and well-protected organizations are regularly the targets of cyberattacks (Verizon, 2025). Automated laboratory operators may be able to avoid some attacks through standard quality control, logging, and cybersecurity efforts (Stouffer et al., 2023; National Institute of Standards and Technology, 2024). However, these efforts should be coordinated between laboratories and proportionate to the risk each laboratory poses.
- Sector scale: The automated laboratory sector remains small, limiting aggregate risk from incidents. However, for intentional misuse, a single high-capability facility could be sufficient.

#### 3.3.3 Malicious actor dynamics

- Cost barriers for malicious biological orders: While establishing legitimate automated laboratory access is expensive, this cost barrier does not apply to cyberattacks or insider threats, which can exploit existing infrastructure without purchasing services.
- Alternative pathways: Traditional routes to biological agents, including university laboratories, do-it-yourself laboratories, or direct equipment acquisition, may currently present fewer barriers. However, as AI-driven cyber capabilities advance (potentially automating the compromise of automated laboratories), or if security tightens for these alternative pathways, this calculus may shift.

## 4 The Automated Laboratory Security Tier (AST) framework

### 4.1 Risk-based classification system

To address the threats described above, we propose the Automated Laboratory Security Tier (AST) framework, which categorizes automated biological laboratories based on the capabilities and biosecurity risks they pose if compromised. The framework also links each tier to proportionate security controls.

The framework assigns facilities with greater latent capability to produce or enhance harmful biological agents proportionately stronger safeguards. Existing biosafety frameworks do not address this. BMBL, the NIH Guidelines, and Select Agent Program Regulations are focused on the agents that laboratories knowingly handle; none account for facilities that work exclusively with low-risk agents but possess latent capability to synthesize harmful pathogens, produce biological toxins, or engineer pathogen-relevant proteins. The AST framework addresses this by classifying facilities not by what they currently handle, but by what they could produce if compromised. For AST-2, this includes two distinct latent capabilities: pathogen synthesis and automated protein engineering, both of which can be exploited through the same access vectors described above.

Our framework uses NIH risk group classifications^1^ (RG1-4) and Department of Health and Human Services (HHS) and U.S. Department of Agriculture (USDA) Select Agents as its foundation for categorizing pathogen severity. We chose risk groups over BSL levels because risk groups classify the pathogen itself (e.g., Ebola is RG4), whereas biological safety levels classify the laboratory environment required to handle it.

Table 1 presents capability definitions and security requirements. Controls are cumulative, meaning each tier implements all lower-tier protections plus additional measures.

The laboratory classification criteria in column 1 of Table 1 give a general framework of how an automated laboratory should be classified. Significant additional work is needed to turn these classification criteria into actionable assessments that automated laboratory operators can use. Initially, laboratories could perform voluntary self-assessments to determine where they fall in the framework. This could shift over time to structured external audits and red-teaming exercises, and eventually include government oversight. We discuss these challenges further in Section “5.1 The innovation-security balance” and in Supplementary Material 3.

The framework comprises three tiers. AST-0 encompasses facilities not equipped to synthesize, modify, or propagate living organisms, viruses, or nucleic acids. These require no special biosecurity measures, beyond routine operations to manage the laboratory. AST-1 includes any facility equipped to work with living organisms, viruses, or nucleic acids that has not been determined to have AST-2 capabilities. This includes facilities assessed as capable of synthesizing only organisms not associated with disease in healthy adult humans (RG1) if fully compromised, as well as facilities not yet assessed. Minimal safeguards are introduced at this tier because even laboratories that cannot independently produce pathogens could serve as one link in a split-order attack across multiple facilities (see section “5.2 Limitations”).

AST-2 includes facilities capable of synthesizing one or more human, animal, or plant pathogens classified as RG2-4 or designated as HHS or USDA Select Agents, if fully compromised. We group RG2-4 together because viral rescue techniques can produce viral pathogens across this range, and we could not find evidence that viral rescue difficulty scales with pathogen severity. While existing biosafety regulations distinguish safeguards by the harms posed by an agent, our framework is different. Since we are classifying laboratories by their latent capability, instead of by the agents they contain, our tiers must align to distinguishable levels of capability. Further research is needed to determine whether additional granularity in latent laboratory capability can be measured, in a way that maps to potential harms. We focus on viral pathogens since these are the most feasible to produce in near-term automated biological laboratories, but the ability to produce any qualifying pathogen would place a laboratory in AST-2.

Automated protein engineering represents a second latent capability that can independently place a facility in AST-2. Recent advances have integrated AI protein design tools with automated platforms, creating closed-loop systems capable of rapidly optimizing biological functions, demonstrated for enzyme activity (Zhang et al., 2025). These capabilities have dual-use potential and could translate to optimization of viral fitness and immune evasion (Wang et al., 2026). A facility lacking full pathogen synthesis capability may still be able to optimize a viral protein for a harmful aim, such as enhanced receptor binding or increased immune evasion, if it has access to appropriate expression systems and directed evolution workflows. Accordingly, the ability to execute automated protein engineering on pathogen-relevant proteins is included as a second AST-2 classification criterion alongside synthesis capability. The controls proposed for AST-2 facilities are likely appropriate for protein engineering risks as well, given that the same access vectors apply; however, additional work may be needed to develop safeguards specific to this threat.

Within AST-2, “Low” and “High”” are formal subcategories that determine the necessary safeguards. AST tiers 0, 1, and 2 classify facilities by latent capability, the most harmful pathogen a facility could produce if fully compromised. The Low/High designation classifies an AST-2 facility along a second axis: operational risk factors that amplify the consequences of compromise. Low represents baseline controls warranted for any AST-2 facility, given that all AST-2 facilities can by definition synthesize harmful pathogens or engineer pathogen-relevant proteins. AST-2 High adds further controls warranted when operational factors increase the likelihood, severity, or reach of a successful compromise.

The operational factors that drive Low/High classification are: accepting customer biological samples (which expands the input vector for malicious orders), shipping biological materials offsite (which expands the egress vector for completed harmful agents and motivates the outgoing-material sequencing required at High), large scale-up capacity (which amplifies the impact of a compromise), and the ease with which the laboratory can execute pathogen synthesis (the simpler the path to harm, the less reaction time defenders have). This list is not exhaustive and may grow as automated laboratory capabilities and operational practices evolve. The specific weighing of these factors, including thresholds for assignment to High, should be standardized through industry-regulator collaboration rather than asserted in this paper.

### 4.2 Framework development and refinement

This AST framework represents a preliminary attempt to systematically categorize automated biological laboratories by their potential biosecurity risks. While grounded in established biosafety principles and informed by expert interviews, the framework requires refinement through real-world application and testing.

First, the capability assessments underlying each tier require validation through systematic evaluation of existing facilities. Our current categorizations are based on theoretical analysis and limited facility information. Thorough testing of what automated laboratories can actually produce under various compromise scenarios remains to be conducted. Red team exercises, carefully designed with appropriate safeguards, could help validate whether our tier boundaries accurately reflect risk levels.

The choice of influenza PR8 as an AST-2 analogue test was informed by expert consultation with a virologist. Horsepox is one example of a virus that has been rescued from synthetic DNA, but one interviewee noted this has proven difficult to replicate in practice. PR8, a mouse-adapted influenza strain, offers a more reproducible capability benchmark while remaining in RG2 and generally requiring only BSL-2 precautions. These two analogue tests could help establish the extent of a laboratory’s latent capabilities, with horsepox as the more challenging test. The third analogue test, engineering binding affinity of a benign protein through automated workflows, has not been validated through expert consultation in the same way as the synthesis tests, and methodology for this test requires further development.

Second, the proposed security controls need field testing to ensure they are both technically feasible and effective without unduly hindering legitimate research. The controls presented here draw from analogous frameworks including NIST Cybersecurity and Operational Technology Frameworks (Ross et al., 2021; Stouffer et al., 2023; National Institute of Standards and Technology, 2024) and IEC 62443 (International Electrotechnical Commission, 2013) for industrial control systems. Their application to automated biological laboratories may reveal domain-specific challenges. Pilot implementations at volunteer facilities could identify practical barriers and necessary modifications.

Finally, the framework must adapt to advancements in AI, cyber, and automation capabilities. Regular reassessment will be essential. We outline potential implementation pathways, including voluntary industry coordination, verification mechanisms, and considerations for mandatory oversight, in Supplementary Material 3. We envision voluntary industry coordination as the near-term primary route, with third-party verification for AST-2 facilities and mandatory requirements held in reserve if voluntary adoption proves inadequate or capabilities advance materially.

## 5 Discussion

### 5.1 The innovation-security balance

There are legitimate concerns that security requirements might stifle innovation. However, appropriate security standards could accelerate development by building public trust and attracting investment. Security measures may also serve industry self-interest. They could help secure valuable intellectual property against theft, and avoid misuse incidents that could trigger heavy-handed ad-hoc regulation. Security infrastructure often has dual benefits: Know Your Customer (KYC) verification procedures support both security screening and customer onboarding. Anomaly detection also supports operations and quality control. The gene synthesis industry demonstrates this dynamic: many providers have established voluntary screening protocols without harming growth or profitability (Carter and Friedman, 2015; Diggans and Leproust, 2019).

Critical infrastructure sectors, including power grids and water utilities, demonstrate that rigorous security frameworks are compatible with managing technologies that present significant risk if compromised. Like automated laboratories, these systems are vulnerable to cyberattacks (Stouffer et al., 2023), and we draw on similar cybersecurity and operational technology security measures being adopted in those industries. Specific governance models from these sectors also informed our framework: the mandatory, FERC-approved North American Electric Reliability Corporation Critical Infrastructure Protection (NERC CIP) cybersecurity standards for the bulk power system (Federal Energy Regulatory Commission, 2008) and the sector-specific Information Sharing and Analysis Centers (e.g., E-ISAC, WaterISAC, BIO-ISAC) that coordinate threat intelligence across providers (Pulivarti et al., 2023; Stouffer et al., 2023). Both also inform the implementation pathways outlined in Supplementary Material 3.

As noted in the introduction, properly governed automated laboratories could enhance overall biosecurity. Centralized, monitored facilities with standardized security controls could be safer in the aggregate than the existing distributed landscape of smaller, manually operated laboratories, and could also reduce hazards to laboratory workers. To achieve this balance, security measures must be proportionate, technically feasible, and developed collaboratively with industry stakeholders.

### 5.2 Limitations

Establishing a new protocol in an automated laboratory still requires significant human effort and tacit knowledge, according to our interviews with laboratory operators. Most laboratory operators and biosecurity experts we interviewed did not believe current automated laboratories have the latent capability to produce pathogens without extensive effort by on-site workers. However, the actual capability of automated laboratories to synthesize pathogens remains untested. Any such testing should use low-risk proxy organisms rather than harmful pathogens, to reduce biosafety risks and the potential of establishing “misuse blueprints.”

Future research priorities should include systematic capability assessments of existing facilities, red-teaming exercises, and development of technical safeguards. One example of such a technical safeguard would be hardware-enforced logging of all protocol executions, sample movements, and equipment operations that cannot be overwritten even if control software is compromised.

Sophisticated actors could chain together multiple lower-tier facilities to achieve higher-tier capabilities. An attacker could orchestrate workflows across several laboratories, each performing legitimate-seeming experiments that collectively accomplish what would require a high-capability facility. For example, separate facilities could synthesize obfuscated DNA fragments, assemble constructs, and produce viruses. Effective response to split-order threats likely requires cross-laboratory data sharing and coordinated screening protocols beyond what voluntary industry coordination can sustain on its own, and would benefit from national-authority involvement (e.g., HHS, or USDA APHIS for animal and plant pathogens) for cross-facility information sharing. Our framework does not fully address this threat, since each laboratory in the chain could be AST-1. Further efforts are needed on cross-laboratory coordination and screening of incoming and outgoing samples.

Some challenges are shared between automated laboratory safeguards and gene synthesis screening. While many sequences of concern are known and identifiable (e.g., the smallpox genome and genes), biological AI models are enabling the design of proteins with limited homology to known pathogens, but the same function. Using these tools, an attacker may be able to redesign a pathogen to evade gene synthesis screening. This challenge applies to our proposal to use nucleic acid sequence screening as a safeguard. We also propose the use of emerging techniques like functional homologue detection, which may soon address this limitation.

### 5.3 Conclusion

Automated biological laboratory capabilities currently appear limited, but rapid advances in automation and AI could enable concerning capabilities in the future. To address these risks, we propose the AST framework. This framework categorizes biosecurity risks based on the potential of an automated laboratory to synthesize pathogens or perform automated protein engineering, and pair each tier with proportionate security controls. These controls are aimed at detecting and preventing compromise through malicious orders, insider threats, and cyberattacks.

Laboratories, industry associations, biosecurity professionals, and policymakers must work together to secure the narrow subset of capabilities relevant to misuse. This engagement is what will allow automated biological laboratories to deliver on their enormous potential.

## Figures and Tables

**FIGURE 1:**
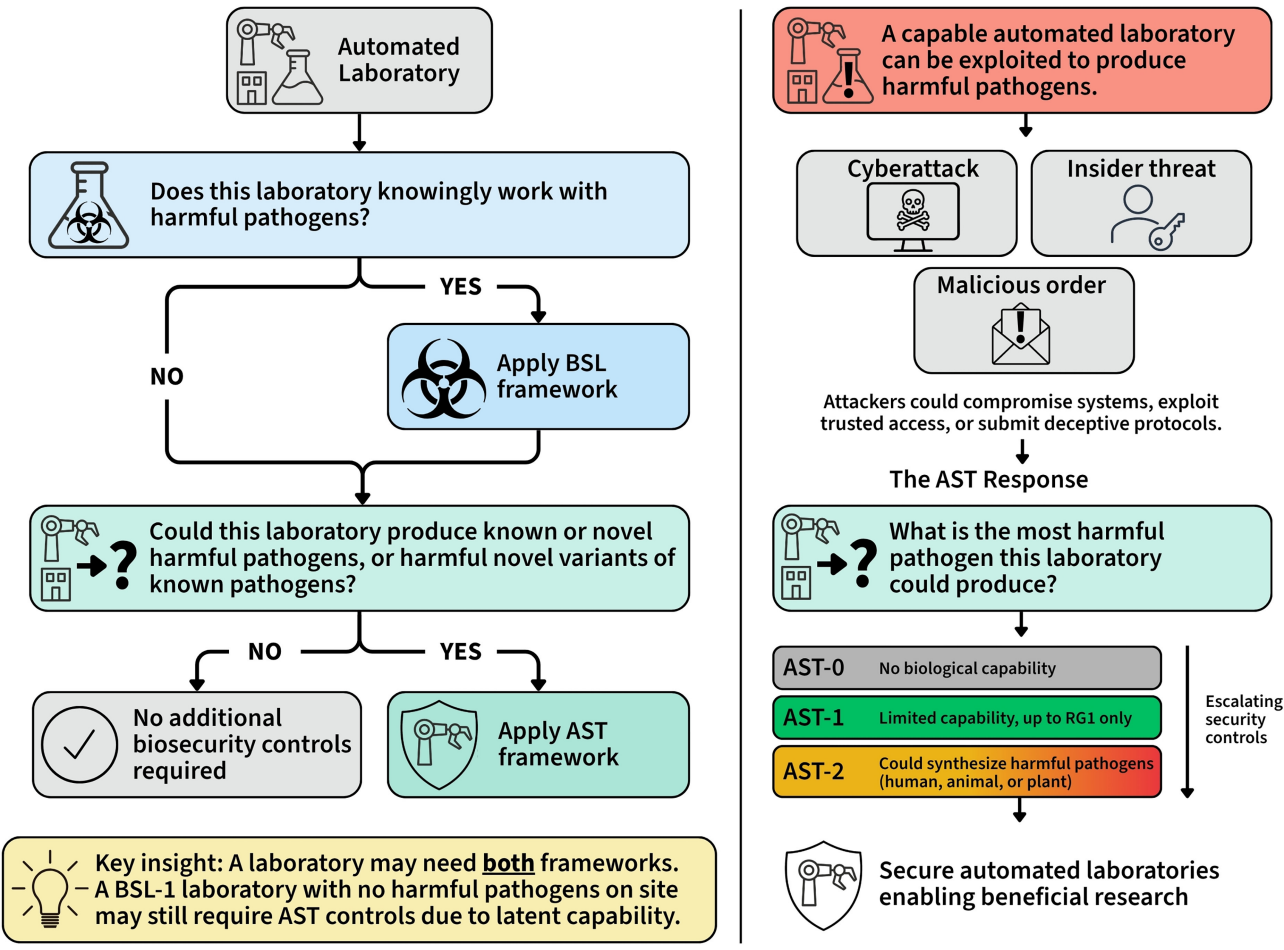
Biosafety and biosecurity decision logic for automated biological laboratories and the Automated Laboratory Security Tier (AST) framework. Decision logic for applying biosafety and biosecurity controls to automated biological laboratories **(left)** and the AST framework response to threat vectors **(right)**. A single facility may require both Biological Safety Level (BSL) and AST controls if it knowingly handles dangerous pathogens and also possesses latent synthesis capability. Risk Group 1 (RG1) refers to organisms not associated with disease in healthy adult humans, as defined by the National Institutes of Health (NIH) risk group classification system. See “Risk-Based Classification System” section for full tier definitions. For clarity, the Figure depicts pathogen synthesis as the illustrative latent capability; the framework also addresses automated protein engineering as a second AST-2 criterion.

## Data Availability

The raw data supporting the conclusions of this article will be made available by the authors, without undue reservation.

## Footnotes

1 The NIH is currently modernizing its biosafety oversight framework (Bhattacharya, 2025) and whether Risk Groups will be retained is uncertain. Future iterations of the AST framework should align with all updated classification schemes.
