# Supplementary material for: Automated Laboratory Security Tiers: a framework for evaluating and mitigating biosecurity risks from latent capabilities
Source: Front Microbiol. 2026 Jul 15;17:1832401. doi: 10.3389/fmicb.2026.1832401 (PMC13416263; doi:10.3389/fmicb.2026.1832401)
Supplement: Supplementary file 1 [file Supplementary_file_1.pdf]

Supplementary Material

S1: Interview methodology

Nineteen semi-structured interviews (45-60 minutes) were conducted from September to December 2025 with 21 participants across three groups:

1. Biosecurity and relevant domain experts, including researchers at policy institutes, government officials, and academics (n=9)
2. Biotechnology industry professionals, including representatives from gene synthesis firms, industry consortia, and biosecurity-focused organizations (n=7)
3. Operators and scientists at four U.S. automated laboratories, including cloud laboratory, biofoundry, and other automated laboratory settings (n=5)

Interviewees were identified through targeted outreach and introductions from initial participants. Some participants had recently transitioned between organizations and spoke from experience at both current and prior affiliations. Inclusion criteria were domain expertise in one or more of: biosecurity policy, automated laboratory operations, cyberbiosecurity, gene synthesis screening, or biotechnology industry practice.

Interviews were conducted on background. Participants were informed that their statements would not be attributed to them or their organizations in any publication arising from this research. For this reason, interview findings are presented in aggregate throughout the manuscript, and identifying details have been omitted or generalized. This approach is standard in policy research involving industry practitioners and government officials, where non-attribution encourages more candid responses. The trade-off is that readers cannot verify the source of specific claims drawn from interviews.

Interview approaches varied by group. Interviews with groups 1 and 2 were exploratory and conversational rather than structured around a fixed question set. We first shared the problem we were working on, of whether automated laboratories might pose a meaningful biosecurity risk. We then invited them to share any relevant perspectives they might have, whether it was on automated laboratories, cyberbiosecurity, virology, or other topics. In early interviews we generally focused on questions such as "What potential risks do you see from automated laboratories?" and "How would you think about making automated laboratories safe?" In later interviews we would share drafts of the Automated Laboratory Security Tier (AST) framework to get their feedback and suggestions for refinement.

The following are the questions we asked operators and scientists at U.S. automated laboratories (group 3). Due to time limitations not all questions were covered in every interview, we prioritized questions based on each individual's expertise and willingness to share details.

1. Can you talk me through your general approach to biosecurity today? How do you think about the risk profile of your lab, and about preventing misuse or accidents?

2. What kinds of checks or screening do you have in place for customers, orders, and experiments?

3. How do you approach decisions about who can access or control safety-critical systems or processes? (i.e., those that, if misused, could produce a dangerous product)

4. How do you think about hiring and managing lab staff, particularly those involved in safety-critical or sensitive work - do you have certain criteria, background checks, or other measures in place? How do you see that evolving in the future?

5. Can you tell me about how you manage cybersecurity, particularly around lab automation and (if any) customer inputs?

6. What practices or frameworks guide your cybersecurity approach (e.g., industry standards, customer requirements)?

7. When you think about lab security or potential risks, do you ever consider scenarios where an adversary could gain substantial control over lab systems, even manipulating logs/QC and safety systems? How do you approach thinking about or preventing those scenarios?

8. What solutions, practices, or guidelines would you find most helpful in making biosecurity and cybersecurity easier or more effective?

9. What federal agencies (if any) would you prefer to be involved, and in what ways?

10. How do you see biosecurity practices evolving as automated labs become more capable and widespread?
