# Supplementary material for: Automated Laboratory Security Tiers: a framework for evaluating and mitigating biosecurity risks from latent capabilities
Source: Front Microbiol. 2026 Jul 15;17:1832401. doi: 10.3389/fmicb.2026.1832401 (PMC13416263; doi:10.3389/fmicb.2026.1832401)
Supplement: Supplementary file 2 [file Supplementary_file_2.pdf]

Supplementary Material

S2: Relevant U.S. Federal Regulations for Automated Biological Laboratories

Automated biological laboratories are subject to multiple federal regulatory frameworks depending on the work they perform. This table summarizes the primary biosecurity- and biosafety-relevant regulations applicable to automated laboratories operating in the United States but is not an exhaustive list.

Agency	Regulation	Citation	Application to Automated Biological Laboratories
**Occupational Safety and Health Administration (OSHA)**			
OSHA	Occupational Exposure to Bloodborne Pathogens	29 CFR 1910.1030	Applies when laboratory personnel work around automated systems handling human blood, tissues, or other potentially infectious materials.
OSHA	Hazard Communication	29 CFR 1910.1200	Requires hazard labeling and safety data sheets for chemicals used in automated workflows, including reagents and solvents.
OSHA	Control of Hazardous Energy (Lockout/Tagout)	29 CFR 1910.147	Applies during maintenance and servicing of robotic equipment to prevent unexpected startup or energy release.
OSHA	Process Safety Management of Highly Hazardous Chemicals	29 CFR 1910.119	Applies if automated laboratories use above-threshold quantities of highly hazardous chemicals.
OSHA	Personal Protective Equipment	29 CFR 1910 Subpart I	Requires appropriate personal protective equipment (PPE) for personnel.
OSHA	Walking-Working Surfaces	29 CFR 1910 Subpart D	Applies to facility layout and maintenance around robotic systems.

**Federal Select Agent Program (FSAP)**			
CDC	FSAP Regulations	42 CFR Part 73	Applies if the laboratory possesses, uses, or transfers agents or toxins of public health concern
Animal and Plant Health Inspection Service (APHIS)	FSAP Regulations	7 CFR Part 331	Applies if the laboratory works with or could produce regulated pathogens of agricultural concern
APHIS	FSAP Regulations	9 CFR Part 121	Applies if the laboratory works with or could produce regulated animal pathogens
**Clinical Laboratory Improvement Amendments (CLIA)**			
Centers for Medicare & Medicaid Services (CMS)	Clinical Laboratory Improvement Amendments	42 CFR Part 493	Applies if the automated laboratory performs clinical diagnostic testing on human specimens.
**Food and Drug Administration (FDA)**			
FDA	In Vitro Diagnostic Products	21 CFR Part 809	Applies if the laboratory develops or manufactures in vitro diagnostic devices or assays.
FDA	Electronic Records; Electronic Signatures	21 CFR Part 11	Applies if the laboratory generates electronic records or uses electronic signatures for FDA-regulated activities. Relevant to audit trail integrity in automated systems.
**Environmental Protection Agency (EPA)**			
EPA	Hazardous Waste Management (Resource Conservation and Recovery Act, RCRA)	40 CFR Parts 260-273	Applies to management and disposal of hazardous chemical and biological waste generated by automated laboratory processes.

EPA	Microbial Products of Biotechnology	40 CFR Part 725	Applies if the laboratory manufactures, imports, or processes new microorganisms for commercial purposes under the Toxic Substances Control Act (TSCA).
EPA	National Pollutant Discharge Elimination System	40 CFR Parts 122-125	Applies if the laboratory discharges wastewater containing biological or chemical pollutants.
**Department of Transportation (DOT)**			
DOT / Pipeline and Hazardous Materials Safety Administration (PHMSA)	Hazardous Materials Regulations (infectious substances)	49 CFR Parts 171-180	Applies when the laboratory ships or receives biological materials classified as infectious substances. Directly relevant to cloud laboratories that accept customer samples and return experimental products.
**Import and Transfer Controls**			
CDC	Importation of Infectious Biological Agents, Infectious Substances, and Vectors	42 CFR 71.54	Applies when importing infectious biological materials into the U.S. Relevant to cloud laboratories receiving samples from international customers.
USDA APHIS	Import/Transit Permits for Animal Pathogens and Vectors	9 CFR Parts 122-130	Applies when importing or transporting regulated animal pathogens. Relevant if automated laboratories receive animal-derived biological materials.
USDA APHIS	Plant Pest Permits	7 CFR Part 330	Applies if the laboratory imports, moves interstate, or releases plant pests, plant pathogens, biological control organisms, noxious weeds, or soil from which microorganisms may be isolated. Relevant to automated

			laboratories working with plants, plant pathogens, or soil.
USDA APHIS	Genetically Engineered Plant Pests	7 CFR Part 340	Applies if the laboratory produces, imports, or moves interstate genetically engineered organisms that may pose plant pest risk. Relevant to automated laboratories performing genetic engineering on plants or plant-associated microorganisms.
**29 CFR 1910 Subpart D**			
Bureau of Industry and Security (BIS)	Export Administration Regulations	15 CFR Parts 730-774	Applies to export of dual-use biological materials, equipment, and related technology. Relevant if automated laboratories serve international customers or transfer materials or technology abroad.

Note: This inventory covers U.S. federal regulations, additional state and local regulations may also apply.
