# Supplementary material for: Automated Laboratory Security Tiers: a framework for evaluating and mitigating biosecurity risks from latent capabilities
Source: Front Microbiol. 2026 Jul 15;17:1832401. doi: 10.3389/fmicb.2026.1832401 (PMC13416263; doi:10.3389/fmicb.2026.1832401)
Supplement: Supplementary file 3 [file Supplementary_file_3.pdf]

Supplementary Material

S3: Implementation Considerations

As described in the main text, the current capabilities of automated biological laboratories are uncertain and the AST framework itself requires validation through real-world application. We therefore outline potential implementation pathways rather than propose a specific timeline. The appropriate approach will depend on how laboratory capabilities evolve and what initial efforts reveal.

Industry Coordination. The near-term priority is establishing voluntary industry self-governance. The International Gene Synthesis Consortium (IGSC) offers a precedent: gene synthesis providers developed shared screening standards before regulatory mandates, and this voluntary coordination has been credited with improving biosecurity without impeding commercial growth (Carter and Friedman, 2015; Diggans and Leproust, 2019). RAND has similarly recommended industry coordination among cloud laboratory operators (Lee and Del Castello, 2024). Participating laboratories could conduct AST self-assessments, share best practices for security implementation, and develop standardized protocols for the controls outlined in Table 1 of the main text. Incident reporting mechanisms and secure channels for sharing threat intelligence across providers would strengthen these efforts. An industry working group or association could also coordinate capability assessments, helping laboratories determine their AST tier through structured evaluation rather than ad hoc self-classification.

Transparency and Verification. Self-reported AST classifications would need independent verification to be credible. As the sector grows and the framework is refined, third-party capability assessments could confirm whether facilities have accurately evaluated their latent capabilities and whether security controls are functioning as intended. The Federal Select Agent Program's inspection model offers one reference point, though the AST framework covers a broader range of facilities, most of which would fall under AST-0 or AST-1 and require minimal oversight. For AST-2 facilities, third-party audits and red-team exercises (as specified in Table 1) would serve both verification and security improvement functions. Assessment results could be reported confidentially to a designated government agency, enabling oversight without publicly disclosing facility capabilities or specific vulnerabilities. The choice of agency and reporting structure would require further deliberation among government and industry stakeholders.

Mandatory Oversight. If voluntary coordination fails to achieve adequate adoption or security outcomes, or if capability advances or security incidents materially change the risk picture, mandatory requirements may become necessary. We do not propose specific triggers or timelines for such a transition. These decisions would need input from industry, government, and biosecurity stakeholders, and would benefit from evidence generated during the voluntary phase on both the feasibility of AST controls and the actual capabilities of existing facilities. Any mandatory framework should preserve the proportionality principle central to the AST approach. AST-0 and most AST-1 facilities should face minimal additional burden, with more demanding requirements concentrated on the small number of facilities whose capabilities warrant them.

International Coordination. Automated laboratories are geographically distributed and accessible remotely, making biosecurity governance of this sector a cross-border problem. A customer in one jurisdiction can submit orders to a cloud laboratory in another, and split-order attacks (described in the Limitations section of the main text) could span facilities in multiple countries. The cross-border, remote-access nature of cloud laboratory operations makes split-order risk particularly amenable to international harmonization: the same digital interfaces that enable cross-jurisdictional misuse make information-based safeguards (customer verification, sequence screening, shared threat intelligence) tractable to coordinate across borders without requiring bilateral treaties or harmonized domestic regulation.

International industry coordination, modeled on the IGSC's Harmonized Screening Protocol adopted by its global membership (International Gene Synthesis Consortium, 2024), could supplement domestic frameworks. The IGSC offers a directly applicable precedent: a voluntary, industry-led standard adopted across jurisdictions in advance of national regulation, credited with raising biosecurity practice across the gene synthesis sector without depending on government mandates (Carter and Friedman, 2015; Diggans and Leproust, 2019). Over time, harmonizing AST tier definitions and security expectations across jurisdictions would reduce the risk of regulatory arbitrage, where threat actors route orders or attacks to facilities in countries with weaker oversight. The appropriate governance structures for this coordination need to be determined.

Adjacent Sectors and Displacement Effects. Security measures focused exclusively on automated laboratories risk displacing threats toward less regulated alternatives. As we note in the main text, traditional routes to biological agents, including university laboratories, do-it-yourself setups, and direct equipment acquisition, may currently present lower barriers than compromising an automated facility. If AST controls raise the difficulty of exploiting automated laboratories while these alternatives remain unaddressed, threat actors will follow the path of least resistance. Laboratory equipment manufacturers and reagent suppliers are part of this picture as well. Equipment capable of supporting pathogen synthesis is commercially available without biosecurity-specific purchase controls. Contract research organizations that perform wet-laboratory work on behalf of clients occupy a similar space to cloud laboratories but may fall outside any automated-lab-specific framework. Addressing these displacement risks is beyond the scope of the AST framework, but biosecurity governance will eventually need to account for them.

Bibliography

Carter, S. R., and Friedman, R. M. (2015). DNA Synthesis and Biosecurity: Lessons Learned and Options for the Future. La Jolla, CA: J. Craig Venter Institute. Available at: https://www.jcvi.org/research/dna-synthesis-and-biosecurity-lessons-learned-and-options-future

Diggans, J., and Leproust, E. (2019). Next Steps for Access to Safe, Secure DNA Synthesis. *Front. Bioeng. Biotechnol.* 7. doi: 10.3389/fbioe.2019.00086

International Gene Synthesis Consortium (2024). Harmonized Screening Protocol v3.0. Available at: https://genesynthesisconsortium.org/wp-content/uploads/IGSC-Harmonized-Screening-Protocol-v3.0-1.pdf

Lee, Y.-C. J., and Del Castello, B. (2024). Robust Biosecurity Measures Should Be Standardized at Scientific Cloud Labs. Available at: https://www.rand.org/pubs/commentary/2024/11/robust-biosecurity-measures-should-be-standardized.html (Accessed September 12, 2025).
